# *Listeria monocytogenes* 10403S Alternative Sigma-54 Factor σ^L^ Has a Negative Role on Survival Ability Under Bile Exposure

**DOI:** 10.3389/fmicb.2021.713383

**Published:** 2021-10-21

**Authors:** Atsadang Boonmee, Haley F. Oliver, Soraya Chaturongakul

**Affiliations:** ^1^Department of Microbiology, Faculty of Science, Chulalongkorn University, Bangkok, Thailand; ^2^Department of Food Science, College of Agriculture, Purdue University, West Lafayette, IN, United States; ^3^Department of Microbiology, Faculty of Science, Mahidol University, Bangkok, Thailand; ^4^Systems Biology of Diseases Research Unit, Faculty of Science, Mahidol University, Bangkok, Thailand

**Keywords:** RNA-seq, *Listeria monocytogenes*, alternative sigma L, negative regulator, bile, stress

## Abstract

*Listeria monocytogenes* is a Gram-positive bacterium causing listeriosis in animals and humans. To initiate a foodborne infection, *L. monocytogenes* has to pass through the host gastrointestinal tract (GIT). In this study, we evaluated survival abilities of *L. monocytogenes* 10403S wild type (WT) and its isogenic mutants in alternative sigma (σ) factor genes (i.e., *sigB*, *sigC*, *sigH*, and *sigL*) under simulated gastric, duodenal, and bile fluids. Within 10min of exposures, only bile fluid was able to significantly reduce survival ability of *L. monocytogenes* WT by 2 logs CFU/ml. Loss of *sigL* showed the greatest bile resistance among 16 strains tested, *p*<0.0001, (i.e., WT, four single alternative σ factor mutants, six double mutants, four triple mutants, and one quadruple mutant). To further investigate the role of σ^L^ in bile response, RNA-seq was conducted to compare the transcriptional profiles among *L. monocytogenes* 10403S *ΔBCH* triple mutant (lacking *sigB*, *sigC*, and *sigH* genes; expressing housekeeping σ^A^ and σ^L^) and *ΔBCHL* quadruple mutant (lacking all alternative sigma factor genes; expressing only σ^A^) strains under BHI and 1% bile conditions. A total of 216 and 176 differentially expressed genes (DEGs) were identified in BHI and bile, respectively. We confirmed that *mpt* operon was shown to be strongly activated by σ^L^. Interestingly, more than 80% of DEGs were found to be negatively regulated in the presence of σ^L^. This includes PrfA regulon and its mediated genes (i.e., *hly*, *hpt*, *inlB*, *clpP*, *clpE*, *groL*, and *inlC*) which were downregulated in response to bile in the presence of σ^L^. This result suggests the potential negative role of σ^L^ on bile survival, and the roles of σ^L^ and σ^B^ might be in a seesaw model prior to host cell invasion.

## Introduction

The foodborne pathogen *Listeria monocytogenes* is a facultative Gram-positive intracellular bacterium that is able to adapt to a board range of habitats. It can survive and grow in a variety of temperatures, and a wide range of pH, osmotic pressure, and high salt (Membre et al., 2005; Goulet et al., 2013; Bevilacqua et al., 2018; Axelsson et al., 2020). This pathogen is the causative agent of listeriosis in human and animals. Although *L. monocytogenes* infections are comparatively rare, the mortality rate could reach up to 30% of clinical cases (NicAogain and O’Byrne, 2016). More importantly, *L. monocytogenes* cannot be underemphasized for it has contributed to huge economic losses in livestock industries, food poisoning leading to death in both animals and human, and abortion in pregnant women (Oliver et al., 2005; Kaur et al., 2007). Following being ingested with contaminated food, *L. monocytogenes* goes through GI passage. It encounters the low pH in stomach and the bile salt and high osmolality in intestinal fluid. Alves et al. have revealed that survival abilities of *L. monocytogenes* under highly adverse conditions mimicking those observed in the gastrointestinal tract are strain-dependent, not serotype-dependent. The survival ability of each strain is affected by previous exposure to stress conditions (Alves et al., 2020). In order to survive and successfully establish infection, *L. monocytogenes* needs to persist exposure to bile, an important antimicrobial component in GI fluid (Olier et al., 2004). It has been reported that *L. monocytogenes* utilizes unique bile resistance mechanisms to survive in the gallbladder (Hardy et al., 2004).

In the host stomach, *L. monocytogenes* has to tolerate acidity. Normally, the pH of the stomach at the fasted-state ranges between 1 and 2 (Dressman et al., 1990; Efentakis and Dressman, 1998). Upon intake of the meal, the fed-state gastric pH increases within a range of 4–7. Once the bacterium enters the intestine, the pH is 6–7.5 (Evans et al., 1988). Little is known about the bactericidal components in the intestinal fluid; however, one crucial component bacteria must cope with is bile. Bile is synthesized by hepatocytes prior to secretion from liver into bile canaliculi. The pH of human hepatic bile is approximately 7.5–8.0 (Begley et al., 2005). Normally, the bile concentration in human gut ranges from 0.005 to 2% (Dawson, 1998). In addition to its role in digestion, bile also has antimicrobial properties. The major components of human bile are bile salt, cholesterol, phospholipid, and various bile acids (Begley et al., 2005; Dowd et al., 2011). It has been shown that *L. monocytogenes* was able to tolerate bovine, porcine, and human bile exposures (Begley et al., 2002; Guariglia-Oropeza et al., 2018). Additionally, *btlB*, *sigB*, *pva*, *prfA*, and *btlA* genes have been considered in bile resistance (Begley et al., 2005). Bile salts are amphipathic molecules that are able to impair lipid-containing bacterial and viral membranes (Dussurget et al., 2002). Moreover, bile salts can also induce DNA damage and RNA secondary structure formation (Begley et al., 2005).

One of the mechanisms a bacterium utilizes to combat cellular stressors is transcriptional regulation of gene expression *via* combinations of alternative sigma factors (σ) and the catalytic core of RNA polymerase. In *L. monocytogenes*, there are four alternative sigma factors (σ^B^, σ^C^, σ^H^, and σ^L^). σ^B^ has been intensively reported as a key factor responsible for *L. monocytogenes* survival under various stress conditions including high osmolality, low or high temperature, low pH, ethanol, and oxidative stresses (Becker et al., 1998; Ferreira et al., 2001; Moorhead and Dykes, 2004; Lungu et al., 2009; Mujahid et al., 2013b). Also, the σ^B^ regulon has been identified as crucial to GI survival. *sigB* deletion mutants show increased susceptibility to bile; however, σ^B^ regulon was not upregulated under exposure to bile pH 5.5 (Guariglia-Oropeza et al., 2018). It is possible that other alternative sigma factors also contribute to bile response. We hypothesized that co-regulation of sigma factors allows *L. monocytogenes* to survive under bile stress and subsequently establish infection. We examined the role of and the co-regulation among alternative sigma factors using single, double, triple, and quadruple mutants. Altogether, we were able to map complex transcriptional regulation by the alternative sigma factor σ^L^ and our results suggest a negative role of σ^L^ in bile stress response.

## Materials and Methods

### Bacterial Strains and Growth Conditions

*Listeria monocytogenes* 10403S and its isogenic single/double/triple/quadruple alternative sigma factor mutant strain were used in this study (Supplementary Table S1). Strains were maintained in brain heart infusion broth (BHI; Difco™, BD, United States) and 50% glycerol stocks at −80°C. They were streaked onto BHI agar plates prior to each experiment, and plates were incubated at 37°C overnight. A single colony was inoculated into 5ml BHI broth at 37°C overnight (16–18h) incubation with shaking (200rpm). Overnight culture was diluted 1:100 into a fresh 5ml BHI broth and incubated at 37°C with shaking to an OD_600_ of approximately 0.4, representing mid-log phase. An aliquot of 500μl of the culture was subsequently passaged in 50ml of BHI broth to generate synchronized cells at mid-log phase before exposure to the simulated bile stress.

### Gastrointestinal Stress Survival Assay

Mid-log phase cells were challenged with simulated gastric, duodenal, and bile fluids independently. The *in vitro* GI fluids were prepared as previously described (Versantvoort et al., 2005). Briefly, 0.5ml of 2X gastric (pH 1.3), 2X duodenal (pH 8.1), or 2× 6% porcine bile (pH 8.2, Sigma, United States) was added to 0.5ml of mid-log phase culture. The challenged cultures were incubated at 37°C with shaking for 10 and 20min, respectively. Survival ability was determined at 0, 10, and 20min after stress exposure (*t*=0, *t*=10, and *t*=20). A 100μl aliquot of the pre-treated control (culture before treatment at *t*=0) and the untreated control (culture with additional 20min incubation) along with *t*=10 and *t*=20 cultures was 10-fold serially diluted in PBS (pH 7.4); 10μl of each dilution was plated onto BHI agar plates for subsequent enumeration. Experiments were conducted in three biological and technical replicates. All survival assays were performed in the absence of complementation strains.

### Statistical Analysis for Survival Assay

Significant differences in survival abilities between *L. monocytogenes* wild type and its isogenic mutants were determined by one-way ANOVA with Dunnett’s *post hoc* test using GraphPad Prism version 9.2.0 (283), GraphPad Software, San Diego, California United States. *p*<0.05 was considered statistically different. To assess which sigma factor has the potent role in bile survival, the listeria strains have been categorized into groups with the presence or absence of each sigma factor. The average log reduction of all strains and replicates in each group was compared using unpaired t test.

### RNA Isolation and DNase Treatment

For gene expression studies, RNA was extracted from mid-log phase cultures after 10-min exposure to 1% porcine bile or BHI (as a control) as previously described with minor modifications (Versantvoort et al., 2005; Pleitner et al., 2014). Briefly, all experiments were conducted in three biological replicates on different days. For each strain and condition, 1ml of BHI or bile-exposed culture was collected and immediately added to ice-cold stop solution of 10% acid-phenol chloroform pH 4.5 (Invitrogen™, United States) in ethanol as outlined previously (Pleitner et al., 2014). DNase treatment was performed using TURBO DNA-*free*™ DNase treatment kit (Invitrogen, United States) according to the manufacturer’s protocol. Total RNA concentration was quantified using NanoDrop 2000c spectrophotometer (Thermo Scientific, Wilmington, DE, USA).

### rRNA Depletion, Library Preparation, and RNA Sequencing

Ribosomal RNA (rRNA) was depleted using Ribo-Zero rRNA removal kit (Epicentre, Madison, WI) following manufacturer’s instruction. Quality of RNA was assessed using the 2,100 Bioanalyzer (Agilent Technology, Santa Clara, CA). Samples with RNA Integrity Number (RIN) score greater than 8.0 were considered acceptable for further library preparation and RNA sequencing. cDNA library preparation was constructed using ScriptSeq v2 RNA-seq Library Preparation kit for Bacteria (Epicentre, Madison, WI). Purification of cDNA and indexed RNA-seq libraries was performed using Agencourt® AMPure® XP kit (Beckman Coulter Inc., Brea, CA). Quantity and quality of the libraries were determined using the 2,100 Bioanalyzer. All experiments were performed in three biological replicates. Sequencing was carried out on a HiSeq 2×100 High Output paired-end, 100bp read at the Purdue University genomics core facility.

### RNA-Seq Analysis

Sequencing reads were mapped against *L. monocytogenes* 10403S (NCBI accession number: NC_017544.1). Reads were aligned and mapped with Bowtie2 and TopHat2 (Langmead and Salzberg, 2012; Trapnell et al., 2012). The cutoffs for percent mapped reads on either sense or antisense strand and percent of rRNA match rate were at least 75% and lower than 0.1%, respectively. Reads were counted by HTSeq-count (Anders et al., 2015). Differentially expressed genes (DEGs) were compared and analyzed in R version 3.3.3 using the package DESeq2 (Love et al., 2014). The analyses were conducted from three replicates of each sample, except wild-type sample in BHI (two replicates were used in analysis). Genes were considered differentially expressed when log_2_ fold change < −1 or>1 (or FC<−2 or>2, representing down- or upregulation, respectively) and adjusted *p*-values <0.05.

### Data Availability Statement

The data sets generated or analyzed for this study were deposited in a public database and can be found under the SRA accession number: PRJNA544468.^1^

For review purposes, RNA-seq data are available at https://drive.google.com/drive/folders/1R3Wv3JN3ICxmY8ScbikALlzjlklfuObi?usp=sharing.

### Gene Set Enrichment Analysis

GOseq package 1.34.1 package for R available from Bioconductor was used to evaluate whether differential expressed genes identified by DESeq2 were enriched for Gene Ontology (GO) terms (Young et al., 2010). This tool allowed us to statistically confirm the specific metabolic pathways that *L. monocytogenes* utilized under BHI and/or bile conditions.

### Quantitative Reverse Transcriptase PCR Validation of RNA-Seq Data

Select differentially expressed genes from RNA-seq results were validated using quantitative reverse transcriptase PCR (qRT-PCR) as previously described with minor modifications (Sue et al., 2004; Pleitner et al., 2014). Target genes used for RNA-seq data validation were (i) *rpoB* and *bglA* as housekeeping genes and (ii) *mptA*, *ilvD*, *ilvC*, *csbD*, and *LMRG_00704* for sigma L function. A list of TaqMan primers and probes designed by PrimerQuest (IDT DNA, Coralville, IA) is shown in Supplementary Table S2. TaqMan probes were synthesized with a 5' 6-carboxyfluorescein (6-FAM) reporter dye and a 3’ ZEN™ dark quencher dye. All reactions were run *via* Rotor-Gene Q (Qiagen, Germany) with the following conditions: 1cycle at 48°C for 30min, 1cycle at 95°C for 10min, followed by 40cycles at 95°C for 15s and 55°C for 1min. Reactions without Multiscribe™ reverse transcriptase (Thermo Fisher Scientific, Wilmington, DE, United States) were run in parallel to account the possible genomic DNA (gDNA) contamination. Threshold cycle more than 35 was considered as undetectable. gDNA standard curve targeting each gene was performed to determine the amplification efficiency. Dilutions of gDNA in 10^7^, 10^5^, and 10^3^ chromosomal copies were used as templates. Transcripts were normalized by a geometric mean of housekeeping genes *rpoB* and *bglA*. Statistical comparisons of gene expression levels among *L. monocytogenes* strains in each condition were performed by unpaired t test, SPSS version 23 (IBM, United States).

## Results

### *Listeria monocytogenes* Survives Well in All Gastrointestinal Fluids Except Bile

**Figure 1:**
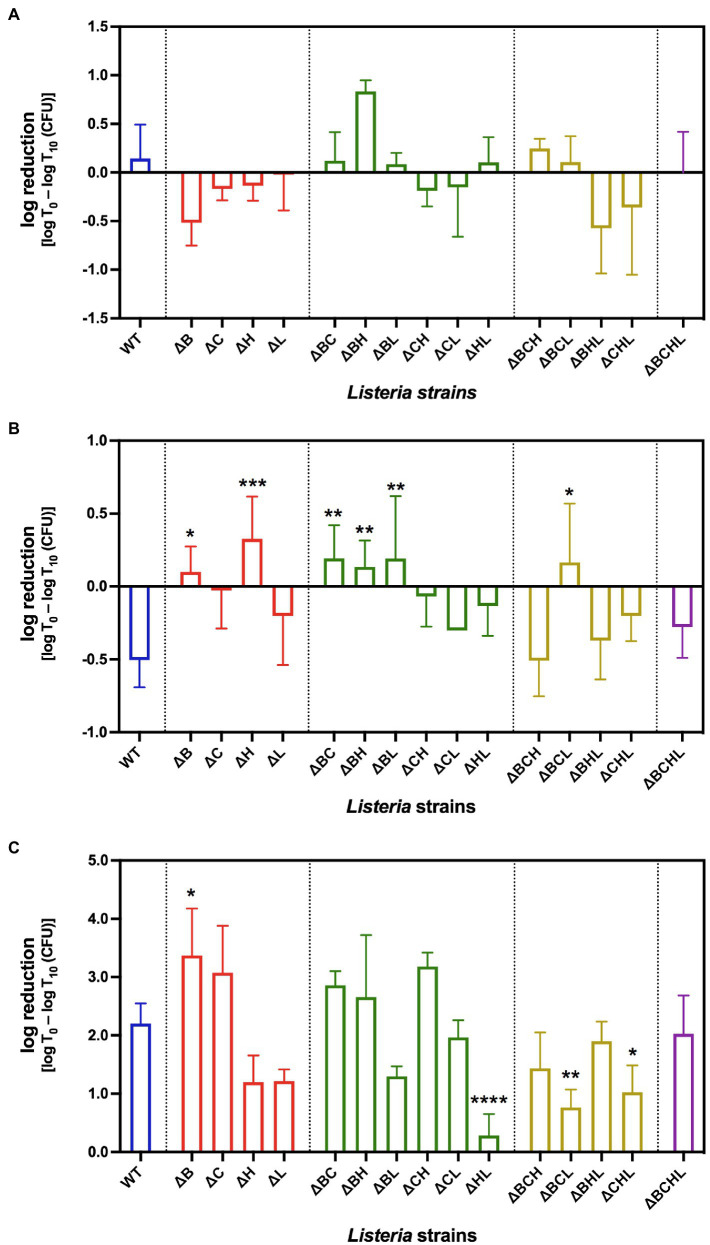
Average log reduction [log T0 – log T10 (CFU)] of *Listeria monocytogenes* 10403S WT (blue), single mutants (red), double mutants (green), triple mutants (yellow), and quadruple mutant (purple) in simulated gastrointestinal fluids: **(A)** gastric fluid, **(B)** duodenal fluid, and **(C)** 6% bile fluid at 10min (T10) post-treatment. *, **, ***, and **** indicate significant difference compared to WT using One-way ANOVA followed by Dunnett’s multiple comparisons test (*p*<0.05, *p*<0.01, *p*<0.001, and *p*<0.0001, respectively).

To determine the ability of *L. monocytogenes* to survive under simulated GI fluids, we exposed the bacteria to simulated gastric, duodenal, or bile for 10 and 20min. Survival abilities at 20min were not statistically different from those at 10min for all strains and conditions. Therefore, the presented data are that of 10min (T10) only. Following 10-min gastric fluid exposures, most of the deletion mutant strains survived similar to wild type, that is, similar levels of log reduction [i.e., log (CFU at T0)−log (CFU recovered at T10)] when compared between WT and mutants (Figure 1A). Among the single mutants, only *ΔB* mutant had a substantially better survival than WT with an increase in numbers during gastric fluid exposure. Among the double mutants, only *ΔBH* double mutant had an immensely higher susceptibility than WT, hence higher log reduction. Among the triple mutants, the presence of σ^C^ in *ΔBHL* vastly increased survival. Interestingly, we did not observe any significant differences between the quadruple mutant (*ΔBCHL*) and the WT when exposed to gastric fluid, suggesting the compensatory effect of housekeeping sigma factor σ^A^ on *L. monocytogenes* survival in gastric treatment.

Following gastric exposure, we next examined survival ability of *L. monocytogenes* under duodenal fluid stress show in Figure 1B. Among the single mutants, significant differences were observed in *ΔB* (*p*=0.0127) and *ΔH* (*p*=0.0003) mutants (i.e., their survival abilities were less than that of the WT). Survival abilities of double mutants, on the other hand, were diverse. While *ΔBC*, *ΔBH*, and *ΔBL* mutants showed significantly higher susceptibilities (*p*<0.01), *ΔCH* and *ΔHL* exhibited lower growth when compared to WT. Among the triple mutants, the presence of σ^H^ in *ΔBCL* mutant resulted in higher susceptibility to duodenal fluid when compared to WT. Similar to gastric treatment, the absence of all four alternative sigma factors resulted in similar survival ability as wild type, suggesting again the compensatory role of σ^A^ under duodenal fluid exposure.

Following gastric and duodenal exposures, we further determined the survival ability of *L. monocytogenes* in simulated bile pH 8.2. Survival of *L. monocytogenes* exposed to simulated bile is shown in Figure 1C. Interestingly, after exposure to simulated bile juice for 10min, *L. monocytogenes* WT decreased by 2 logs CFU/ml. Approximately 3-log reduction was observed at 10-min exposure for *ΔB* and *ΔC* single mutants compared to the corresponding pre-treated culture. The log reduction in *ΔB* was significantly higher than that of wild type (*p*=0.0268). The absence of σ^H^ or σ^L^ alone in *ΔH* and *ΔL* strains resulted in better survival than WT. In addition, double deletions of *sigH* and *sigL* resulted in even more pronounced ability to survive in synthetic bile for *ΔHL* (*p*<0.0001). In contrast to *sigL* deletion, the absence of σ^C^ in *ΔBC* and *ΔCH* resulted in a higher decline in the bile exposure survival experiment compared to WT. Nevertheless, two triple mutants (*ΔBCL* and *ΔCHL*) survived significantly better than WT (*p*<0.05). As observed in gastric and duodenal fluid treatment, the quadruple mutant survived similar to wild type. Collectively, we suggest that σ^B^ and σ^C^ play positive roles in response to bile exposure, while σ^H^ and σ^L^ likely play negative roles on *L. monocytogenes* survival under bile treatment. We further focused on the effect of each alternative sigma factor on *L. monocytogenes* survival in simulated bile. Among the alternative sigma factors, σ^L^ showed the greatest significant effect on *L. monocytogenes* survival abilities in synthetic bile (*p*=0.000).

### Interaction of σ^H^ and σ^L^ Decreases Resistance to Bile

**Figure 2:**
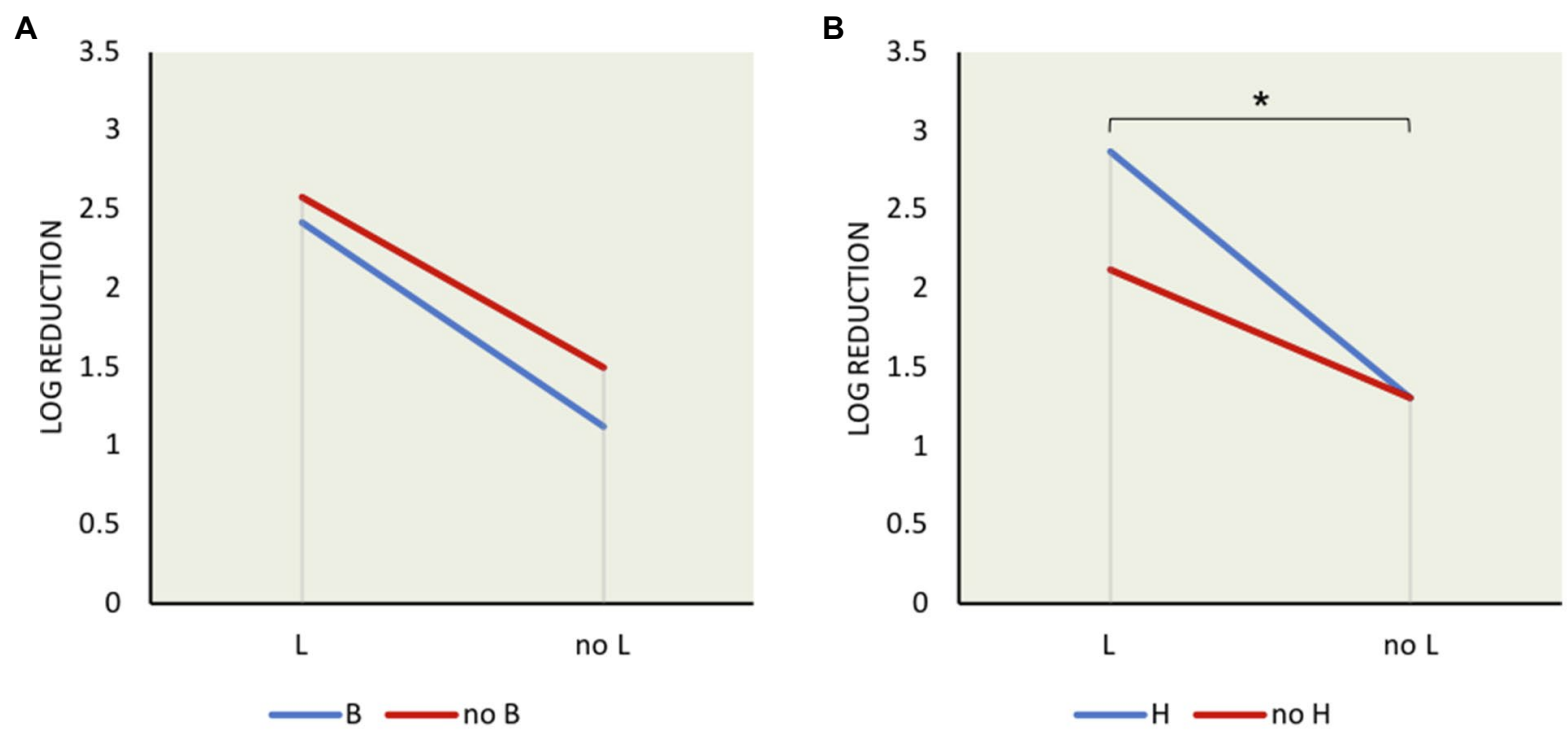
Mean interaction plots of *L. monocytogenes* log reduction after exposure to 6% bile for 10min showing the influence of the presence and absence of σ factors: **(A)** σ^B^ and σ^L^ interaction **(B)** σ^H^ and σ^L^ interaction. *indicates significant difference between two indicated factors using *t* test (*p*<0.05).

Although σ^L^ showed the greatest effect on *L. monocytogenes* survival in bile, the effects of other sigma factors were also determined. In order to decipher the interaction among sigma factors and the redundancy effect, we performed mean interaction plots showing the mean of log reductions from the presence and absence of each sigma factor compared with one another as shown in Figure 2. The effect of σ^B^ was compared in strains with σ^B^ (i.e., wild type, *ΔC*, *ΔCH*, and *ΔCHL*) and without σ^B^ (i.e., *ΔB*, *ΔBC*, *ΔBL*, and *ΔBCHL*). The presence or absence of σ^B^ though could not significantly induce bile resistance if σ^L^ was present (Figure 2A), and the presence of σ^B^ together with σ^L^ slightly increased survival ability in bile treatment. Contradictory, loss of σ^L^ greatly increased survival ability in presence or absence of σ^B^ while having σ^B^ only was better than absence of both. In other words, σ^B^ increases bile resistance but this effect is dampened in the presence of σ^L^. The negative role of σ^L^ is stronger and could mask the positive role of σ^B^ on bile resistance. On the other hand, survival ability was significantly low in the presence of both σ^H^ and σ^L^ in comparison with the absence of both, *p*<0.05 (Figure 2B). Loss of σ^H^ increased the survival ability of *L. monocytogenes* only while σ^L^ still functioned. This finding suggests that σ^H^ and σ^L^ interacted synergistically to reduce resistance to bile. It could be suggested that σ^L^ plays a negative role on *L. monocytogenes* survival under bile since the presence of σ^L^ was always associated with lower survival. In addition, *L. monocytogenes* survived greater when σ^L^ was absent.

### σ^L^ Downregulates Negative Regulator Genes Under 1% Bile Exposure

To determine the potential negative regulation of *L. monocytogenes* σ^L^ under simulated bile exposure, we compared the RNA-seq transcripts from *ΔBCH* strain exposed to bile to that without bile exposure. Since *ΔBCH* harbors σ^L^ as the only remaining alternative sigma factor, the identified regulon should indicate the effect of σ^L^ alone or the effect of σ^L^ in combination with the housekeeping sigma factor σ^A^ under bile exposure. With this bile exposure and non-exposure comparison, we identified 57 genes that were differentially expressed under bile in *ΔBCH*. Eighteen out of 57 genes showed higher transcripts under bile exposure, while 39 genes were downregulated as listed in Supplementary Table S3. Among 39 downregulated genes, two known negative regulators, *ctsR* and *hrcA*, showed lower transcripts in bile condition. CtsR, a transcriptional repressor, controls the class III heat shock genes Clp ATPase including *clpC*, *clpP*, and *clpE* (Nair et al., 2000). HrcA has been found to repress expression of *dnaK* and *groSL* operons that contain *hrcA-grpE-dnaK-dnaJ* and *groS-groL* genes, respectively (Woodbury and Haldenwang, 2003). Moreover, the tryptophan biosynthesis-associated genes (*trpDEG*) were also downregulated.

### Differentially Expressed Genes Under Loss of σ^L^ Condition

To assess the effect of the absence of σ^L^, we further compared RNA-seq data between *ΔL* mutant grown in BHI and exposed to 1% bile. In this analysis, we found 84 genes were differentially expressed as shown in Supplementary Table S4. Of these, 36 genes were considered upregulated under bile treatment. The highest fold changes (FC) were observed in *clpE* gene and *ilv* operon ranging from 4 to 8 FC. *clpE* encodes ATP-dependent Clp protease, ATP-binding subunit ClpE, while the *ilv* operon is involved with valine biosynthesis. Universal stress response *uspA2* gene was also significantly upregulated under bile exposure.

Conversely, 48 genes were downregulated in the absence of σ^L^ under bile exposure. Among them, approximately 3–4 FC was observed from *LMRG_00042*, *LMRG_01298*, and *LMRG_01900*. These genes are involved with glycerol catabolic pathway, nucleoside triphosphatase YtkD, and mannitol-specific phosphotransferase system (PTS), respectively. In addition, some transporter genes, such as *LMRG_02716* and *LMRG_01818*, as well as flagella biosynthesis-associated gene *flhF*, were also downregulated during bile exposure.

### σ^L^ Seemingly Plays a Negative Role in Gene Expression

To determine the role of σ^L^ in gene regulation, RNA-seq data from *ΔBCHL* and *ΔBCH L. monocytogenes* mutant strains grown in BHI were compared. *ΔBCHL* lacks all alternative sigma factors and only harbors the housekeeping σ^A^, while *ΔBCH* mutant represents the sole presence of alternative σ^L^ which could co-ordinate or compete with the housekeeping σ^A^. RNA-seq data comparison of *ΔBCHL* and *ΔBCH* allowed us to evaluate the regulation of σ^L^ with minimal redundancy of alternative sigma factors σ^B^, σ^C^, and σ^H^. Under BHI only condition, *ΔBCHL* and *ΔBCH* transcript comparison revealed 216 genes (7.63% of 2,828 protein coding genes) that were significant differentially expressed (Supplementary Table S5). Interestingly, a total of 174 genes (approximately 80% of 216 genes) were negatively regulated under σ^L^ and only 42 genes were identified as positively regulated by σ^L^.

Among the positively regulated genes shown, expression levels of *mptC* and *mptD* encoding for mannose-specific PTS components IIC and IID, respectively, were notably differentially expressed (Supplementary Table S5). They were highly expressed with more than 300 FC, suggesting strong positive regulation by σ^L^. *LMRG_02229* and *LMRG_02348* encoding for cellobiose-specific PTS component IIC and putative regulator of the mannose operon, *manO*, respectively, showed increased expression levels up to 23 FC. Moreover, other mannose-specific PTS component genes including *mpoABCD* operon were also positively regulated under σ^L^. Markedly, genes in glutamate decarboxylase in GAD systems, *gadD2* and *gadT2*, showed higher transcripts as well.

Besides these positively regulated genes, 174 genes were defined as negatively regulated by σ^L^, of which the *LMRG_00091- LMRG_00094* operon encoding fructose-specific PTS components showed lower transcript levels as low as −39 FCs. *LMRG_01669*-*LMRG_01672* involved in myo-inositol degradation, *LMRG_02570* encoding a cellobiose-specific PTS component, and *LMRG_01248-LMRG_01249* encoding galactitol-specific PTS components also showed decreases in gene expression. Moreover, *plcA*, *plcB*, *actA*, and *csbD* were also negatively regulated under σ^L^.

### Role of σ^L^ in Bile Exposure

**Figure 3:**
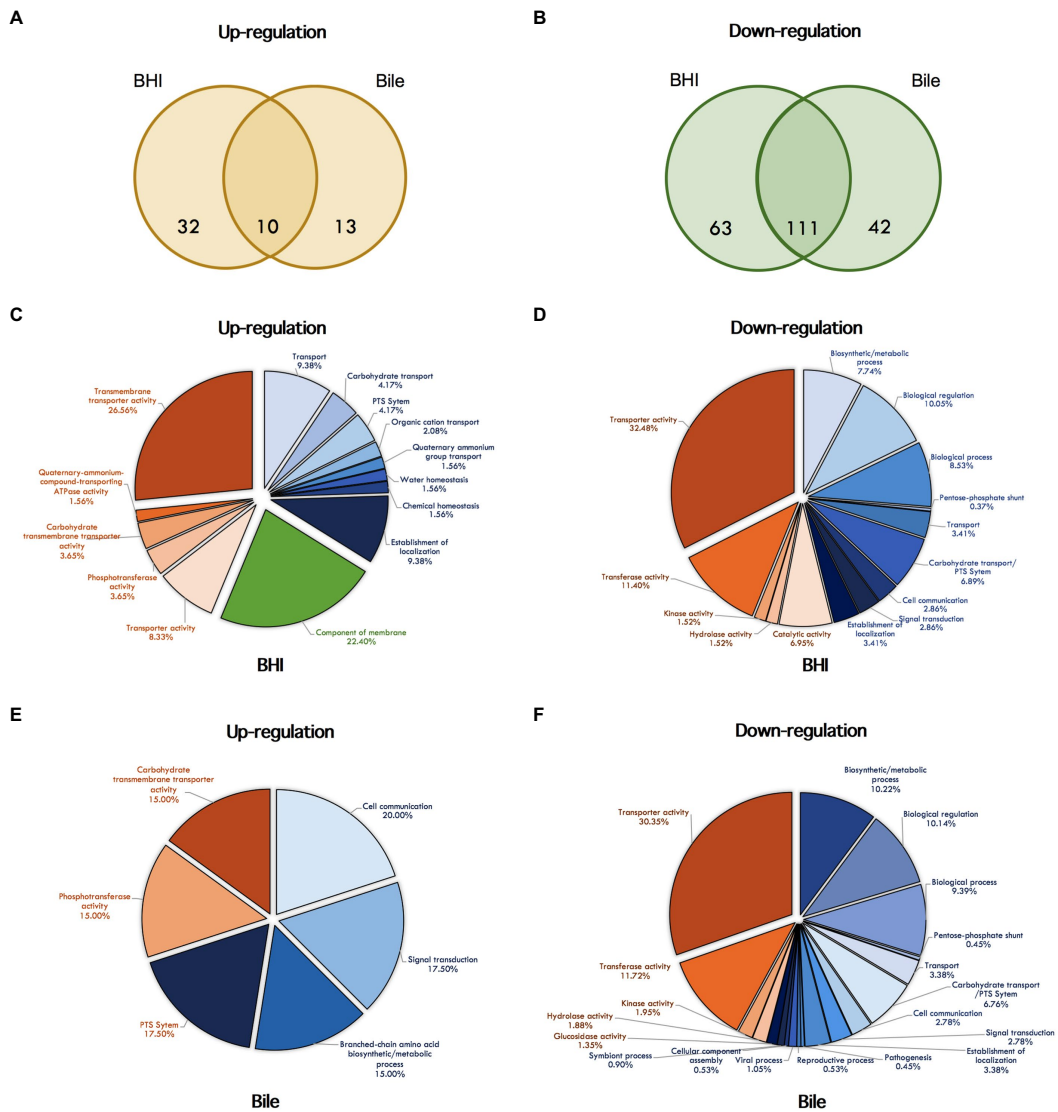
Venn diagrams showing the overlap of differentially expressed genes under σ^L^ regulation with significantly higher **(A)** and lower **(B)** levels. Differentially expressed genes in BHI and bile conditions are shown in the left and the right circles, respectively. The number indicated in the circle represents the number of differentially expressed genes in each condition. Pie chart showing GO term enrichment under σ^L^ regulation in BHI and bile. The GO term categories enriched among up- and down-regulated genes under BHI are shown in **(C)** and **(D)**, under bile are shown in **(E)** and **(F)**. Pie color indicate categories: blue, biological process; red, molecular function; green, cellular components.

In order to determine the role of σ^L^ regulation during bile exposure, we further analyzed RNA-seq data from *ΔBCH* and *ΔBCHL L. monocytogenes* mutant strains in bile. We identified 176 genes that were differentially expressed in bile condition, while 23 and 153 genes showed higher and lower transcripts, respectively (Supplementary Table S6). Later, we compared the differentially expressed genes from bile and BHI conditions to identify differentially expressed genes in bile only. Among 23 upregulated genes, we found 13 genes were highly expressed under bile condition only. Among 153 downregulated genes found during bile exposure, only 42 genes showed lower transcripts (FC<−2) in bile condition, but not in BHI. A Venn diagrams showing the overlap of transcripts with significantly higher (upregulated) and lower (downregulated) levels in BHI and bile exposure are demonstrated in Figures 3A,B, respectively.

Among the 13 upregulated genes under bile exposure, *LMRG_02230* had the highest FC at 13; it encodes PTS mannose transporter subunit IIA. *LMRG_01481* and *LMRG_01482* encoding Rrf2 family transcriptional regulator and membrane protein, respectively, showed higher expression levels at 3–4 FC. *leuC*, *ilvA*, *ilvC*, *yneA*, and *cggR* were also listed in this category. *leuC* encodes 3-isopropylmalate dehydratase large subunit in leucine biosynthesis pathway. *ilvA* and *ilvC* are threonine-dehydratase- and ketol-acid-reductoisomerase encoded genes in valine biosynthesis and isoleucine biosynthesis, respectively. *yneA* encodes the sugar-binding component ABC transport protein, while *cggR* encodes central glycolytic genes regulator.

We had considered the role of σ^L^ in negative regulation in BHI since more than 80% of differentially expressed genes had reduced transcripts. By comparing BHI and bile RNA-seq data, up to 86% of differentially expressed genes were downregulated, of which only 42 genes were identified in bile exposure conditions. Interestingly, *prfA* and its mediated genes *hly*, *hpt* and *inlB*, as well as virulence-associated genes *clpP*, *clpE*, *groL*, and *inlC*, were also downregulated in this condition. However, other PrfA-dependent virulence-associated genes *plcA*, *plcB*, and *actA* showed lower transcripts in both conditions.

In addition to differentially transcribed genes, we identified 19 and 52 GO terms that were enriched under σ^L^ regulation in BHI with positive and negative manners, respectively, supporting the major role in negative regulation by σ^L^ (Figures 3C,D). Of these, GO terms for the components of membrane and the transport system, including PTS system, organic, carbohydrate, quaternary-ammonium-compound transporting system, are overrepresented among the upregulated genes under σ^L^ suggesting a role in transport system. In addition, among 52 GO terms identified, several biological and cellular processes and various transport systems, such as PTS system, transmembrane transporter, cation-proton symporter, and disaccharide transport, were found to be enriched among downregulated genes under σ^L^ regulation in BHI. A total of eight and 64 GO terms were found to be overrepresented among up- and downregulated genes under bile exposure, respectively (Figures 3E,F). As predicted, we found that the GO terms associated with carbohydrate transport including PTS system and branched-chain amino acid biosynthetic process were enriched among upregulated genes upon bile exposure. Apart from these GO terms, the GO terms for multiple cellular processes, carbohydrate, alcohol, and glycerol metabolic process, several transport systems, PTS system, and disaccharide transport were found to be enriched among downregulated genes under bile exposure. Surprisingly, the GO terms associated with viral process, virion assembly, and reproduction were overrepresented among downregulated genes.

### Loss of σ^L^ Induced Gene Expression

We investigated the effects of the loss of σ^L^ in gene expression control. RNA-seq data from *L. monocytogenes* wild type and *ΔL* mutant in both BHI and bile were compared to evaluate the differential gene expression. In BHI, we observed 218 differentially expressed genes. Of these, 191 genes were upregulated in the absence of σ^L^ (Supplementary Table S7). However, 27 (12.39%) genes were downregulated in the absence of σ^L^ in BHI condition. Moreover, a total of 266 genes were differentially expressed, and 218 (81.95%) and 48 (18.4%) genes showed higher and lower transcripts in bile exposure condition, respectively (Supplementary Table S8). In contrast to the previous comparison between *ΔBCH* and *ΔBCHL* grown in BHI showing 80% of differentially expressed gene negatively regulated by σ^L^, this comparison between WT and *ΔL* under BHI showed a higher proportion (87.61%) of positively regulated gene by σ^L^. This result confirmed the negative role of σ^L^ in gene expression since loss of σ^L^ could upregulate some of downregulated genes shown in *ΔBCH* and *ΔBCHL* comparison. For example, *LMRG_02569* encoding cellobiose-specific PTS IIB component was shown to be negatively regulated under σ^L^ by −9 FC; however, it had higher expression by 16 FC when in *ΔL* mutant. Beta-glucosidase encoded gene *LMRG_02568* was expressed at low level in *ΔBCH* mutant but its transcript was increased to as high as 14.5 FC in the *ΔL* mutant. This suggested that deletion of *sigL* upregulated genes that were suppressed or downregulated. Among the upregulated gene in *ΔL* background, *LMRG_00091* gene, identified as σ^L^ negatively regulated gene, was also upregulated as high as 61 FC.

**Figure 4:**
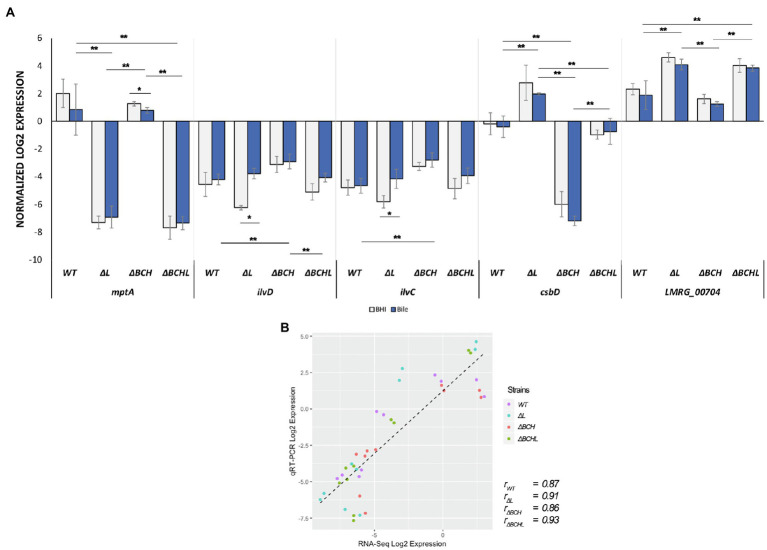
Validation of differential expressed genes (DEGs) by TaqMan qRT-PCR. **(A)** Transcript levels were quantified in, *mptA*, *ilvD, ilvC, csbD*, and *LMRG_00704* in wild type; *ΔL; ΔBCH; and ΔBCHL* quadruple mutant. Transcript levels are expressed as relative to WT after normalization to geometric mean of housekeeping genes; *rpoB* and *bglA*. Blue histograms represented *L. monocytogenes* strains exposed to simulated 1% bile for 10min as compared to its BHI control indicated in grey. Experiments were performed in biological triplicates. **(B)** Pearson correlation coefficients (*r*) of expression levels accounted from RNA-seq and qRT-PCR data in the wild type (purple), *ΔL* (blue), *ΔBCH* (red), and *ΔBCHL* (green). *indicates significant difference in bile treatment compared to BHI using unpaired t-test (p<0.05) and **indicates significant difference among strains within the same condition (p<0.05) using One-Way ANOVA.

**Figure 5:**
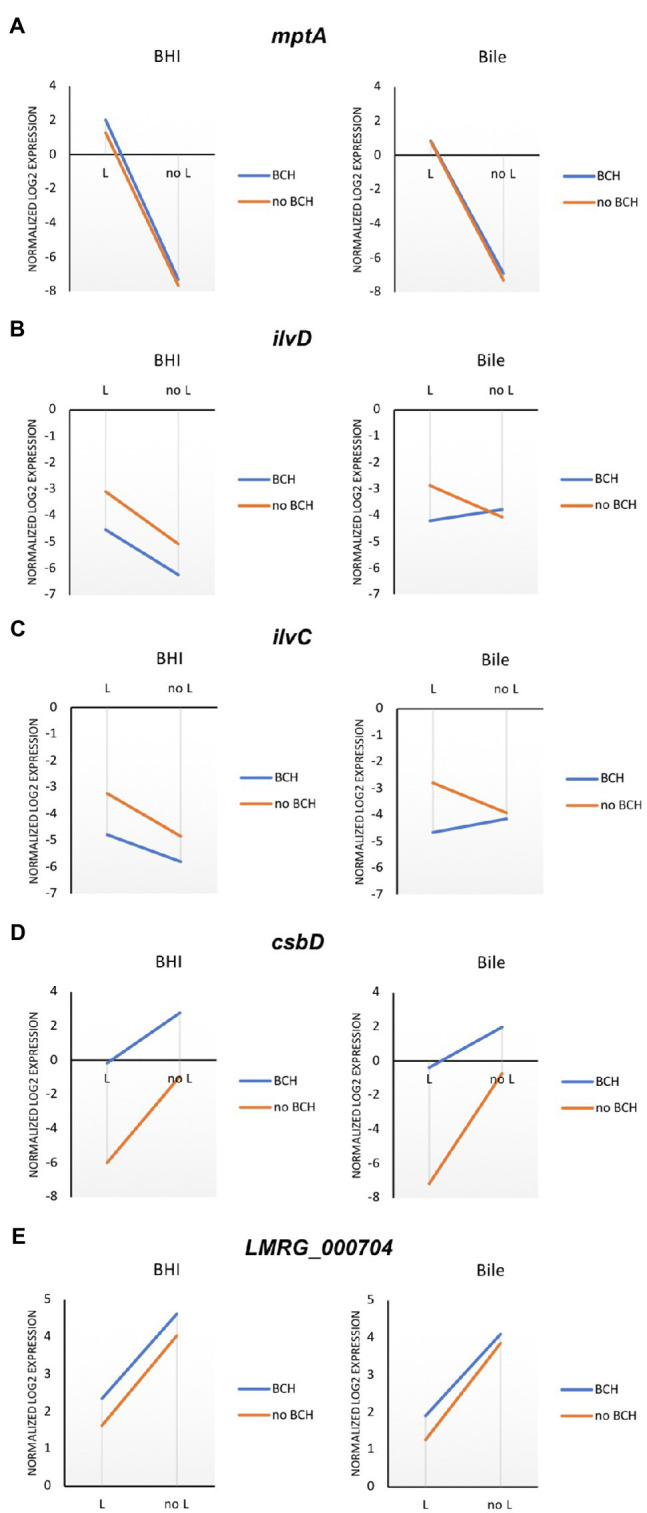
Mean interaction plots of **(A)** mptA, **(B)** ilvD, **(C)** ilvC, **(D)** csbD, and **(E)** LMRG_000704 expression expression levels accounted from wild type, *ΔL*, *ΔBCH*, and *ΔBCHL* in BHI (left) and bile exposure (right) by qRT-PCR. Blue line indicates the presence of σ^B^, σ^C^, and σ^H^ in the conditions, while the orange line represents the strains without those sigma factors.

To identify potential reporter genes for σ^L^ activities, selected sets of σ^L^-dependent genes were verified in qRT-PCR assays. We confirmed the positive and negative role of σ^L^ using differential expression of *mptA* and *csbD*, respectively (Figure 4A). The expression level of *mptA* in *ΔBCH* is similar to wild type but decreased in *ΔL* and *ΔBCHL*, supporting the positive role of σ^L^. Bile is not involved in *mptA* transcription since there is no correlation between the presence and absence of σ^L^ with or without bile (Figure 5A). In contrast, the *csbD* and *LMRG_00704* transcripts are significantly lower in the presence of σ^L^ alone in *ΔBCH* (*p*<0.05); however, their transcripts are highly expressed in *ΔL*. Therefore, *csbD* and *LMRG_00704* are negatively regulated by σ^L^. Bile does not influence the level of *csbD* and *LMRG_00704* as bile does not significantly alter the level of transcripts of these genes (Figures 5D,E). The *ilv* operon (*ilvD-ilvC*) is also upregulated in the presence of bile in *ΔL* (Supplementary Table S4), further confirming that *ilv* operon is negatively regulated under bile exposure. The interaction plot also shows that bile exposure can change the direction of *ilvD-ilvC* expression compared to BHI (Figures 5B,C). Hence, it can be confirmed that bile could upregulate *ilvD-ilvC* expression in the absence of σ^L^. Overall, for gene expression levels of these selected genes, we have shown that RNA-seq and qRT-PCR are well correlated with Pearson correlation coefficients (*r*) of expression levels in the wild type, *ΔL* and *ΔBCH* and *ΔBCHL* mutant at 0.87, 0.91, 0.86, and 0.93, respectively (Figure 4B).

## Discussion

Our data showed that simulated GI fluid, in particular bile fluid, could reduce survival ability of *L. monocytogenes* under high pH mimicking the bile in gallbladder. However, simulated gastric and duodenal fluids could not significantly reduce cell survival. Upon low pH exposure during gastric fluid stress (pH 1.3), we could not observe the reduction of *ΔB* mutant, in contrast to other studies showing that *ΔB* mutant had impaired survival in acidic culture at pH 2.5 compared to its wild type (Ferreira et al., 2003). This conflicted finding might be explained by the difference in exposure time between studies. The *ΔB* mutant was exposed for an hour to acidic pH, while, in our experiment, the incubation time was 10min which could lead to lower CFU reduction. In addition, using an organic acid (acetic acid) is more harmful to bacterial cells than inorganic acid (HCl) used in our system compared to other finding (Werbrouck et al., 2009). The gastric fluid ingredients used in our assay were different from others; therefore, σ^B^ might play a limited role in this gastric stress and time exposure. We also did not observe reduction in *L. monocytogenes* in simulated duodenal fluid. It might be due to lack of key bactericidal enzymes mixed in our duodenal fluid; the enzymes used in our assay were lipase and pancreatin. Lipase typically functions in the digestive system, processing dietary lipids and transport. Pancreatin, produced by pancreas cells, is a combination of digestive enzymes including amylase, protease, and lipase. Its major role is digesting sugars, proteins, and processing fats. Thus, releasing bile from the gallbladder is important not only for digestive system functionality but also as a defensive mechanism against pathogens in the intestine (Boyer, 2013).

A very small number of genes were found to be regulated by σ^L^ in previous studies. According to proteomic analysis, only two proteins were positively regulated by σ^L^ (Mujahid et al., 2013a). Based on our transcriptomic analysis, 42 genes were positively regulated in normal BHI condition. Of these, two of them were consistent to the proteomic study (Mujahid et al., 2013a), which are mannose-specific PTS component gene *mptA* and acetolactate synthase encoding gene *alsS*. *mptA* was not positively regulated in our study; however, genes under its regulon, *mptC* and *mptD*, were positively regulated. Therefore, we assumed that *mptA* was positively regulated by σ^L^ since they were located in the same operon. Unexpectedly, none of the positively regulated genes identified in this study were found in previous microarray analyses (Chaturongakul et al., 2011). This may be due to the cell phase difference of *L. monocytogenes* used. While *L. monocytogenes* grown to log phase was used in our experiment, stationary phase cells were analyzed in a previous assay. In addition to the difference in cell phase, different strains were used in the analyses. In microarray study, *ΔL* was used in comparison with WT (Chaturongakul et al., 2011), whereas in our study, *ΔBCH* and *ΔBCHL* were compared in order to identify σ^L^-dependent genes which turned out to be consistent with the previous proteomic study (Mujahid et al., 2013a). Among the positively regulated genes, we also identified that *mpoABCD* operon which encoded mannose-specific IIABCD component was positively regulated by σ^L^. Arous et al. (2004) have proposed by *in silico* analysis that this *mpoABCD* operon might be regulated by σ^L^. However, Raengpradub et al. (2008) have shown that σ^B^ upregulated the transcription of this operon as well. Therefore, it suggests that operon *mpoABCD* is under co-regulation of σ^B^ and σ^L^. The crosstalk between *mpt* and *mpo* has been demonstrated; however, cross-regulation is not fully understood (Arous et al., 2004). In addition, σ^L^ has previously been shown to contribute to cold, high salt, and organic acid stresses since the impaired growth was observed in the *ΔL* mutant when compared to its isogenic WT (Raimann et al., 2009). However, only one of the cold and NaCl adaptation genes, *oppA*, was shown to be significantly different between *ΔL* and WT under cold stress, while *cspD* and *clpP* did not show statistical difference under 3% NaCl exposure. It is also interesting that our data showed *clpP* one of the negatively regulated genes under σ^L^.

It is likely that σ^L^ negatively regulates a large number of genes, which is concordant with protein analysis (Mujahid et al., 2013a). Among 216 downregulated genes under σ^L^ control identified in both BHI and bile, 27 genes were coherent with 56 proteins identified by proteomic study. We found only two genes that were consistent with previous microarray assays which were *lmo2340 (LMRG_01503)* and *lmo0345 (LMRG_00036*; Chaturongakul et al., 2011). We found that more than 80% of differentially expressed genes were negatively regulated by σ^L^. The role category function of σ^L^ was overrepresented by transport, binding protein, and energy metabolism. Of these, we could only confirm the negative regulation of σ^L^ in *csbD* and possibly *LMRG_02283*. It was clearly shown that in the absence of σ^L^ in *ΔL* mutant, *csbD* transcription was significantly induced. In contrast to the presence of σ^L^ in *ΔBCH* background, where a significant reduction of *csbD* transcript was observed. It was previously shown that *csbD* is a σ^B^-dependent gene (Hain et al., 2008). Nevertheless, we observed the induction of *csbD* in *ΔL* compared to wild type. If it were solely under σ^B^, the level of expression would remain as the wild type. Therefore, we suggested that *csbD* is positively regulated by σ^B^ but negatively regulated by σ^L^. We could not confirm the role of σ^L^ in *LMRG_00091* transcription, though we observed a similar expression pattern to *csbD*, but not significantly different. Interestingly, some virulence-associated genes, (e.g., *actA*, *plcA*, and *plcB*) were downregulated by σ^L^. The association between σ^L^ and virulence is yet to be confirmed. It is possible the negative regulation by σ^L^ is in an indirect manner.

In agreement with our previous study, bile mediated higher production of *ilvC* gene (*ilv* operon) in *L. monocytogenes* 10403S (Boonmee et al., 2019). Based on our current RNA-seq data, *ilvC* was also higher expressed in *ΔBCH* when compared to that in *ΔBCHL* under bile condition suggesting the positive role of σ^L^. We also highlighted that *ilvC* expression level in *ΔBCH* was significantly increased in comparison with that of WT (Figure 4A). In addition, *gadT2* was predicted in Boonmee et al. (2019) to be controlled by alternative sigma factors, that is, significantly higher expression in WT when compared to *ΔBCHL*. We confirmed that *gadT2* was positively regulated by σ^L^ since it showed higher transcripts in both BHI and bile conditions (Supplementary Tables S5 and S6).

Due to a large number of genes negatively regulated by σ^L^, we hypothesized its negative regulation of virulence-associated genes under bile exposure. *prfA*, *inlB*, *inlC*, and *hly* were downregulated by σ^L^ under bile treatment. PrfA, a master virulence regulator, is tightly regulated and has been shown to be positively regulated by σ^B^ (Kazmierczak et al., 2006; Ollinger et al., 2009). We have observed the downregulation of *prfA* itself as well as part of its regulon (*hly*, *hpt*, *inlB*, *clpP*, *clpE*, *groL*, and *inlC*) in response to 1% basic bile (pH 8.2). It was concordant with previous microarray study and with our RNA-seq study showing the whole PrfA regulon was repressed under bile exposure (Quillin et al., 2011; Boonmee et al., 2019). In contrast, Guariglia-Oropeza et al. (2018) reported upregulation of PrfA regulon but not *prfA* itself upon exposure to acidic bile (pH 5.5). It is suggested that the alteration of bile pH could contribute to diverse regulation and transcription levels since pH was considered as a stimulus as well. In addition, other factors such as strain, serotype, and lineage could vary members of bile stimulon (White et al., 2015). Nevertheless, the interplay between downregulation and upregulation and of PrfA by σ^L^ and σ^B^ suggests the transcription network switch between listerial adaptation during GI passage prior to host cell invasion.

## Conclusion

Altogether, to finetune expression of genes under stress exposure, the *L. monocytogenes* 10403S expression systems need to find their new balance. Excessive expression either RNA or protein might be noxious to the cells. The absence of σ^L^, including single, double, and triple mutants, comparatively increases resistance to bile compared to WT suggesting its negative role on bile survival. We have shown that, under bile exposure, a number of genes are negatively controlled by σ^L^. Interestingly, the major virulence factor, PrfA, is identified as negatively regulated by σ^L^. Therefore, the functional roles and the underlying mechanisms in which σ^L^ and σ^B^ co-mediate PrfA expression are to be further investigated. The positive role of σ^B^ and the negative role of σ^L^ support their adaptive roles to finetune expression of survival genes important for bile persistence.

## Data Availability Statement

The datasets generated or analyzed for this study were deposited and can be found under the SRA accession number PRJNA544468 (https://www.ncbi.nlm.nih.gov/sra/PRJNA544468).

## Author Contributions

HO and SC conceived the study. AB performed the experiments and analyzed the data. AB, HO, and SC drafted the manuscript. All authors contributed to the article and approved the submitted version.

## Funding

The study was supported by Office of the Higher Education Commission (Thailand) for Talent Mobility Program (to SC). HO is partially supported by USDA-NIFA Hatch project IND010930.

## Conflict of Interest

The authors declare that the research was conducted in the absence of any commercial or financial relationships that could be construed as a potential conflict of interest.

## Publisher’s Note

All claims expressed in this article are solely those of the authors and do not necessarily represent those of their affiliated organizations, or those of the publisher, the editors and the reviewers. Any product that may be evaluated in this article, or claim that may be made by its manufacturer, is not guaranteed or endorsed by the publisher.

## Footnotes

^1^ https://www.ncbi.nlm.nih.gov/sra/PRJNA544468
